# Amorphous Silicon Oxynitride-Based Powders Produced by Spray Pyrolysis from Liquid Organosilicon Compounds

**DOI:** 10.3390/ma14020386

**Published:** 2021-01-14

**Authors:** Honorata Osip, Cezary Czosnek, Jerzy F. Janik, Jakub Marchewka, Maciej Sitarz

**Affiliations:** 1 Faculty of Energy and Fuels, AGH University of Science and Technology, Mickiewicza 30, 30-059 Krakow, Poland; osip@agh.edu.pl (H.O.); janikj@agh.edu.pl (J.F.J.); 2 Faculty of Materials Science and Ceramics, AGH University of Science and Technology, Mickiewicza 30, 30-059 Krakow, Poland; jmar@agh.edu.pl (J.M.); msitarz@agh.edu.pl (M.S.)

**Keywords:** methylsiloxanes, spray pyrolysis, nitridation, silicon oxynitrides, oxygen contents

## Abstract

Silicon oxynitrides (SiO_x_N_y_) have many advantageous properties for modern ceramic applications that justify a development of their new and efficient preparation methods. In the paper, we show the possibility of preparing amorphous SiO_x_N_y_-based materials from selected liquid organosilicon compounds, methyltrimethoxysilane CH_3_Si(OCH_3_)_3_ and methyltriethoxysilane CH_3_Si(OC_2_H_5_)_3_, by a convenient spray pyrolysis method. The precursor mist is transported with an inert gas or a mixture of reactive gases through a preheated tube reactor to undergo complex decomposition changes, and the resulting powders are collected in the exhaust filter. The powders are produced in the tube at temperatures of 1200, 1400, and 1600 °C under various gas atmosphere conditions. In the first option, argon Ar gas is used for mist transportation and ammonia NH_3_ gas serves as a reactive medium, while in the second option nitrogen N_2_ is exclusively applied. Powder X-Ray Diffraction (XRD) results confirm the highly amorphous nature of all products except those made at 1600 °C in nitrogen. SEM examination shows the spheroidal particle morphology of powders, which is typical for this method. Fourier Transform Infrared (FT-IR) spectroscopy reveals the presence of Si–N and Si–O bonds in the powders prepared under Ar/NH_3_, whereas those produced under N_2_ additionally contain Si–C bonds. Raman spectroscopy measurements also support some turbostratic free carbon C in the products prepared under nitrogen. The directly determined O- and N-contents provide additional data linking the process conditions with specific powder composition, especially from the point of view of oxygen replacement in the Si–O moieties formed upon initial precursor decomposition reactions by nitrogen (from NH_3_ or N_2_) or carbon (from the carbonization of the organic groups).

## 1. Introduction

Silicon nitride-based materials find numerous applications in modern technology due to their excellent chemical, thermal, and mechanical properties. Such powders and ceramics replace materials that cannot resist high temperatures or strong electrical and magnetic fields [1]. Ceramic materials of this type have also shown to have new prospects in medicine [2,3]. Specifically, oxynitride ceramics (SiO_x_N_y_) in the base system Si–O–N are technologically attractive because they possess good mechanical strength that mostly originates from crosslinking made by trivalent nitrogen atoms replacing divalent oxygen in the glass-type of phase (amorphous but not necessarily rigid structure) typical for these materials [4,5]. The incorporation of nitrogen leads to a significant increase in the elastic modulus, hardness, and tenacity of the glass. In this regard, nitrogen incorporation, for instance, into melted borosilicate glasses has been reported as limited [6,7]. To overcome such limitations, the glass should be melted under a reducing atmosphere, or complex ceramics systems such as Y–Si–Al–O–N should be applied [7].

Oxynitride glasses can be prepared by several routes. Nitridation by bubbling ammonia through sodium-calcium-silica melts was a method that allowed the introduction of small amounts (<0.5 wt %) of nitrogen into silicate glasses [8]. Another currently the most studied route is melting metal oxides, silicon dioxide, and nitride compounds, yielding glasses with nitrogen contents typically up to ca. 30 equivalent % [8,9]. Quite a new synthesis method enabling high concentrations of nitrogen up to 65 equivalent % is melting together a pure metal, silicon dioxide, and silicon nitride [8,10]. In this method, the mixture is melted under a nitrogen atmosphere and the modifier introduced into the glass is a selected metal (or metal hydride) instead of a metal oxide. The silicon oxynitride powders may be produced in reactions of gaseous SiO with nitrogen [11,12,13], by carbothermal reduction of silica in nitrogen atmosphere [14] or by amorphous silica nitridation with ammonia [15].

The spray pyrolysis method, also called the aerosol-assisted synthesis method, allows for processing suitable liquid organosilicon precursors into powder products. This method has already been used in our group to prepare pure and composite nanopowders of silicon oxycarbide glasses (SiO_x_C_y_) from selected oxygen-containing organosilicon precursors in a single stage process under Ar [16]. By applying an additional pyrolysis in the second stage, nanometer-sized SiC or C/SiC composite powders could be prepared depending on the type of precursor [17,18,19,20]. In general, such an approach dwells on the complex thermal decomposition changes of a precursor under a neutral gas atmosphere and probable inter-reactions of evolving by-products. The important aspect of spray pyrolysis is that due to relatively short and limited residence times of particles in the high temperature zone of reactor, the decomposition reactions are kinetically controlled and, usually, afford a mixture of by-products. A follow-up pyrolysis of such by-products at suitable high temperatures favors stable products, often in the particle nanosized range, that are produced via thermodynamically driven reactions. The application of a suitable gas atmosphere, neutral or reactive, helps in directing some of these reactions towards a target product.

The goal of this study was to examine an alternative route by spray pyrolysis to the most commonly applied routes for the preparation of the amorphous powders of silicon oxynitride. Herein, data are presented on such powders prepared by spray pyrolysis applied to selected oxygen-bearing liquid organosilicon precursors. The products were characterized with scanning electron microscopy SEM, powder XRD, FT-IR, and Raman spectroscopy. The oxygen and nitrogen contents in the powders were directly determined with an inert gas fusion analyzer.

## 2. Materials and Methods

**Figure 1:**
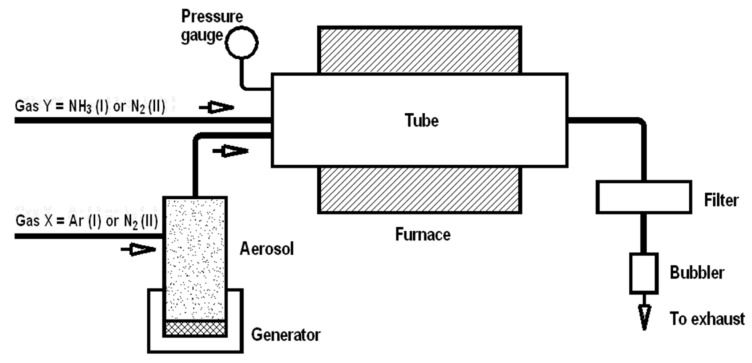
Schematics of experimental set-up for in-flow spray/aerosol pyrolysis of methyltrialkoxysilanes under various gas atmospheres. Note, an Ar/NH_3_ gas mixture is applied in option I, whereas exclusively N_2_ gas is applied in option II.

**Synthesis.** Two organosilicon molecular compounds, methyltrimethoxysilane (MTMS) CH_3_Si(OCH_3_)_3_ (Aldrich, St. Louis, MS, USA) and methyltriethoxylsilane (MTES) CH_3_Si(OC_2_H_5_)_3_ (Aldrich, St. Louis, MS, USA) were used as starting materials in spray synthesis, and details of the method were described previously [16,17,18,19]. The experimental set-up was composed of a pneumatic spray generator with the output connected to a ceramic tube reactor and an exhaust nylon filter attached to the reactor’s exit, as shown in Figure 1. A 1000 mm long electric furnace was used to heat the alumina tube reactor (76 mm ID) to selected temperature levels. The precursor mist was transported by gas through the preheated tube reactor to undergo complex changes, and the resulting powders were collected in the exhaust filter. The powders were produced in the reactor at temperatures of 1200, 1400, and 1600 °C under diverse gas atmosphere conditions. In the first option, argon gas (2 L/min) was used for mist generation and transportation together with added NH_3_ (2 L/min), which served as a reactive medium, while in the second option N_2_ was exclusively applied both for mist generation (2 L/min) and transportation (2 L/min). For convenience, the former case will further be quoted as NH_3_ atmosphere to underline the reactive component in the Ar/NH_3_ gas mixture. Mild oxidation of the samples in air was performed in a muffle furnace at 700 °C in air for 1 h.

**Characterization.** All powders were characterized by FT-IR spectroscopy (Nicolet 380, Thermo Electron Corp., Waltham, MA, USA) using KBr pellets containing about 1 mg of samples. The XRD analysis of the powders was done by Empyrean diffractometer (PANalytical, Malvern, UK), Cu Kα source. The morphology of the selected powders was examined with scanning electron microscopy (SEM) (Hitachi, Tokyo, Japan, model S-4700). The Raman measurements were conducted on a Horiba Jobin Yvon LabRAM HR micro-Raman spectrometer (Horiba, Northampton, UK), excitation wavelength 532 nm, and beam intensity 10mW. The oxygen and nitrogen contents were directly determined with an ONH 836 elemental analyzer (Leco Corporation, St. Joseph, MI, USA).

## 3. Results and Discussion

Precursor molecules transported in an inert gas flow through a pre-heated reactor undergo thermally induced complex decomposition changes that are dependent on the precursor’s chemical make-up and in particular the stabilities of the functional groups. In the system of interest, which is based on such elements as silicon, oxygen, nitrogen, carbon, and hydrogen among various potential groups, the most stable to thermal breaking is the Si–O bond (ca. 450 kJ/mol). This can be compared with the energies of the Si–C (ca. 360 kJ/mol), C–C (ca. 350 kJ/mol), C–O (ca. 360 kJ/mol), and C–H (410 kJ/mol) bonds. Due to the short residence times of the particles in the reactor of the order of several seconds, the kinetically controlled by-products will contain species with the strongest bonds like in SiO_2_ or SiO_x_C_y_/SiO_x_N_y_ but also, possibly, elemental carbon from the decomposition/carbonization of hydrocarbon groups. Hydrogen, some oxygen and some carbon may be removed from the system in the form of volatile species, whereas it is anticipated that silicon will be converted to specific solid by-products by re-forming of stable Si–O, Si–C or Si–N bonds. In this regard, the application of ammonia in one of the process options introduces a significant change in reaction conditions compared with, practically, a neutral nitrogen gas atmosphere at all temperature levels. First, ammonia is known to react at increased temperatures with carbon and carbon-bearing compounds towards volatile products and, therefore, is expected to reduce the amounts of solid elemental carbon in the system up to the point of complete carbon removal. Second, at suitably high temperatures ammonia is prone to react with the in situ formed SiO_x_C_y_ towards SiO_x_N_y_ and to, eventually, by complete O-by-N replacement, form the silicon nitride Si_3_N_4_. These ammonia-mediated reactions are significantly facilitated by ammonia decomposition at increased temperatures, notably above 700 °C, towards very reactive in statu nascendi formed nitrogen and hydrogen molecules, accompanied by their respective atomic species, with a net result of ammonia becoming a very efficient nitridation medium. Additionally, these changes are facilitated by carbothermal reduction processes wherein elemental carbon reacts with oxygen in the Si–O-containing groups to form gaseous carbon oxides, which can further be associated with either remaining carbon or available nitrogen atoms reacting to form Si–C or Si–N bonds, respectively. This means that the presence of free elemental carbon in the decomposing precursor will be advantageous for nitridation reactions of ammonia with the Si–O fragments towards SiO_x_N_y_ [21,22]. In the case of a nitrogen atmosphere, the thermochemistry is expected to be similar at lower temperatures to afford in specific cases the powders of SiO_x_C_y_, SiC, SiO_2_, and/or free C no significant reactions with N_2_. Interestingly, the carbothermal reduction may end up with forming elemental silicon according to a simplified reaction Si–O + C → Si + CO if there are no available C or N atoms present to bind to silicon. At high temperatures of the order of 1600 °C, nitrogen gas may show some nitridation reactivity, although short reaction times of spray pyrolysis will limit its extent. It is instructive to note that, independently from the extent of nitridation reactions in the powders from the spray pyrolysis stage, there is a practically simple way to increase it, if needed, by applying an additional pyrolysis under ammonia at suitably high temperatures.

**Figure 2:**
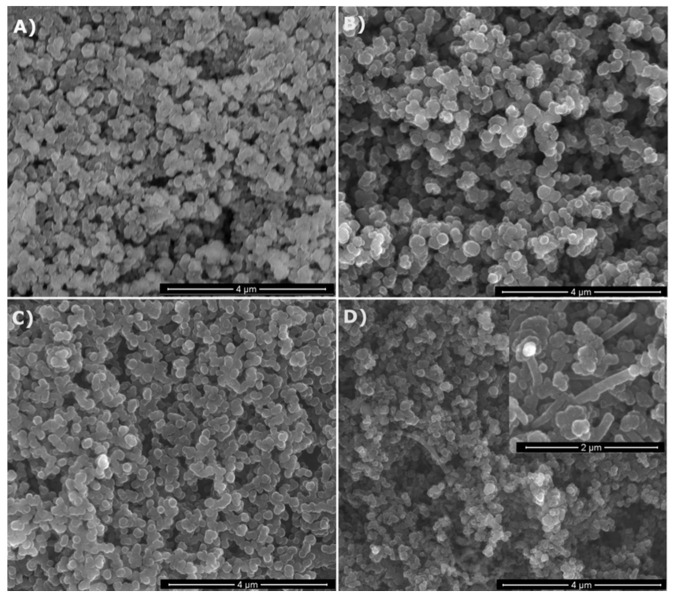
SEM images of selected powders prepared from methyltrimethoxysilane (MTMS): (**A**)—1200 °C/NH_3_, (**B**)—1200 °C/N_2_, (**C**)—1600 °C/NH_3_, (**D**)—1600 °C/N_2_ (note higher magnification of the inset).

The as-prepared products both from MTMS and MTES were all free-flowing powders with different colors depending on the applied gas atmosphere. The powders prepared in NH_3_ were creamy-white, whereas those made in N_2_ were grayish-black. They all possessed the spheroidal particle morphology typical for spray pyrolysis, as seen on the representative SEM images shown in Figure 2 for various powders made from MTMS. The particles were quite uniform in size and occasionally tended to be partly aggregated. No significant differences in morphology were observed among the samples. Nevertheless, in the case of the powder prepared at 1600 °C in N_2_ (Figure 2D, inset) some needle-like moieties (whiskers) were occasionally spotted. The formation of such moieties is generally thought to proceed via the participation of species in the gas phase. As seen in Figure 2D, the extent of whisker formation was limited and rather peripheral under the applied conditions.

**Figure 3:**
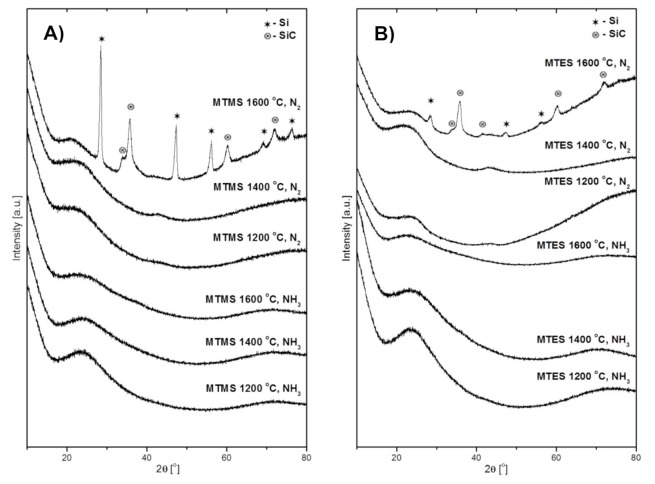
The XRD patterns of powders produced by spray pyrolysis at different conditions. (**A**)—from MTMS, (**B**)—from MTES.

The XRD patterns of the powders produced from MTMS and MTES are presented in Figure 3A,B, respectively. The first three patterns at the bottom of both figures are for the powders prepared in NH_3_ atmosphere and the next three, at the top of the figures, are for those made in N_2_ atmosphere. It can be seen that all of the powders prepared in ammonia were highly amorphous regardless of the temperature and the kind of precursor. Similarly, the powders prepared in the nitrogen flow at 1200 °C and 1400 °C were amorphous as well. It is worth pointing out that the broad features at 2θ ca. 26° (stronger) and 45° (weaker) are characteristic of various amorphous carbon materials [23] whereas the peak at 25° may have a component for amorphous silica SiO_2_ [24,25] with both these materials feasible to be formed in the systems. Rather drastic changes are observed in the case of both powders made at 1600 °C in nitrogen. The diffraction patterns of these powders both from MTMS and MTES include well developed peaks for two crystalline components. The peaks at 2θ ca. 28.4°, 47.3°, 56.1°, 69.1° and 76.4° can be indexed as belonging to the cubic phase of elemental silicon Si (ICDD 00-027-1402), while the peaks at ca. 35.6°, 60.0°, and 71.8° satisfactorily match the cubic polytype of silicon carbide β-SiC (ICDD 00-029-1129). The shoulder at 34.1° is assigned to characteristic stacking faults in β-SiC that are frequently observed in nanocrystalline β-SiC made from various chemical precursors [26,27].

The formation of silicon carbide is expected to occur in this temperature range via the carbothermal reduction of the Si–O-bearing fragments with free carbon and with both species evolved from precursor thermal decomposition, i.e., Si–O + 2C → SiC + CO. In the areas with local carbon deficiency, the reduction would proceed differently, i.e., Si–O + C → Si + CO, resulting in the formation of elemental silicon that is indeed observed (also, see the Raman data below). Both these solid-state reactions appear to take place to various extents in the systems. In this regard, the carbon-related effect/carbothermal reduction would explain the lower proportion of elemental Si under ammonia (compare the relevant top patterns in Figure 3A,B)—the application of ammonia by its efficient reactions with carbon at increased temperatures would limit carbon availability for the deeper carbothermal reduction of Si–O towards SiC. The evolution of more intense and narrow peaks of β-SiC in the MTMS-derived powder compared with the MTES case suggests more favorable crystallization conditions in the former. This may be related to the different elemental composition of the precursors and their effect on the make-up of products. The Si:O:C ratio is 1:3:4 in MTMS (methyltrimethoxysilane) and 1:3:7 in MTES (methyltriethoxysilane). The initial higher content of carbon in MTES results, likely, in a higher (remaining after somewhat competing reactions with Si–O and ammonia) free carbon content in the produced powder. In this regard, our free carbon content analysis performed by mild oxidation of the samples made in nitrogen showed varying carbon amounts, with the MTMS-derived powder having 21 wt % and the MTES-produced powder having 51 wt %. As frequently observed for crystallization phenomena in ceramic composite systems, the higher free carbon content in the latter hampers the crystal growth of other components via the thinning effect, yielding smaller β-SiC crystallites (relatively broader diffraction peaks).

In concluding remarks on the XRD study, the different outcome of reactions in ammonia and in nitrogen, especially at 1600 °C, can be linked to the chemically active role of the former. The ammonia may be a source of reactive nitrogen not only in reactions with carbon species to remove free carbon from the system (as volatile by-products) and prevent silicon carbide formation, but also in prevailing nitridation reactions with Si–O fragments towards silicon oxynitride SiO_x_N_y_.

**Figure 4:**
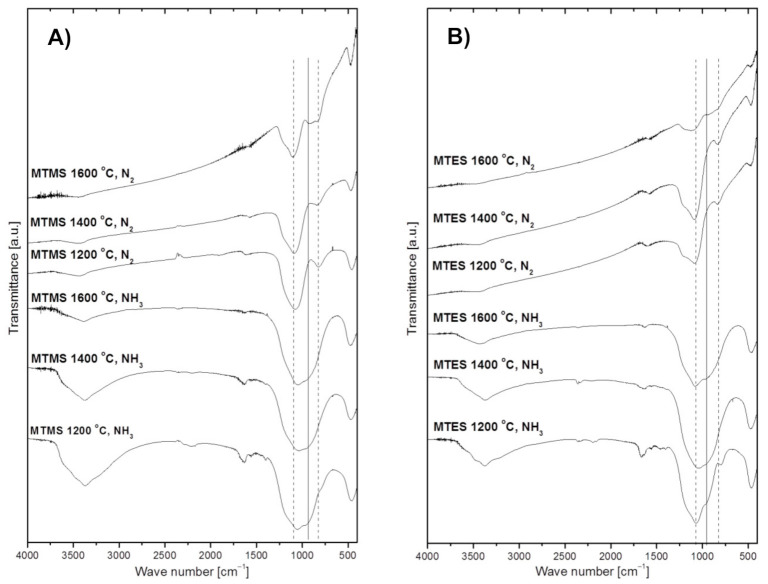
FT-IR spectra of powders produced by spray pyrolysis at different conditions. (**A**)—from MTMS, (**B**)—from MTES. The vertical dashed lines crossing frequent peak positions for Si–O bonds (left line at 1070 cm^−1^) and Si–C bonds (right line at 820 cm^−1^) as well as the solid line for Si–N bonds (middle line at 950 cm^−1^) are guides for eye, only.

The FT-IR spectra of powders prepared from MTMS and MTES are shown in Figure 4A,B, respectively. In general, all spectra display non-specific broad features due to post-reaction adsorbed water and, likely, ammonia in the suitable cases. The water-specific bands are seen at ca. 3430 cm^−1^ (H–O–H asymmetric and symmetric stretch contributing to the broad band in this region) and 1640 cm^−1^ (H–O–H bending mode) [28,29]. The water originates either from adsorption of H_2_O vapor being a nitridation by-product in ammonia (e.g., from a simplified reaction of Si–O + NH_3_ → Si–N + H_2_O + ½ H_2_), which takes place on powders collected on the filter, and/or it is adventitiously adsorbed from the air during KBr pellet preparation (used here for powders made in both gas atmospheres). In this regard, it is worth noting that the relatively more intense water-related bands in powders made in ammonia, which support the abundance of water in these cases. Additionally, there are also spectral features consistent with some adsorbed ammonia in powders made in an Ar/NH_3_ atmosphere, namely, the evolving small intensity peak at ca. 3370–3380 cm^−1^ (N–H degenerate stretches) superimposed on the water band and the corresponding very small intensity band at ca.1560–1580 cm^−1^ (N–H deformation mode), the latter being clearly separated from the relevant bending mode of water at 1640 cm^−1^. These ammonia-related bands can be convincingly discerned for both 1200 °C-derived powders, whereas the low intensity bending mode is apparently below the detection limit for the 1400 °C and 1600 °C-prepared products. Finally, a weak shoulder at ca. 3650–3700 cm^−1^ on the broad water band is consistent with a share of surface hydrogen-bound silanol Si–OH groups formed, for instance, via hydrolysis of the primary surface Si–NH_2_ groups from nitridation (e.g., Si–NH_2_ + H_2_O → Si–OH + NH_3_). All the discussed non-specific features and the species behind them are consistent with the actual chemical and physical circumstances in the systems, however, from quantitative point of view they are considered of secondary significance in this study. With this in mind, the remaining major bands are for us sample-specific and yield important information about the species present in the powders. In all cases, there are only a few bands that show up in the chemically feasible bond mode ranges. Notably, there are no bands in the range 2900–2980 cm^−1^ characteristic of C–H stretching vibrations in hydrocarbon groups, which supports the efficient decomposition-assisted reaction chemistry of both organosilicon precursors towards inorganic solid products. For Si–O species, the stretching and bending vibrations are expected and seen in the 1060–1080 cm^−1^ range and at 470 cm^−1^, respectively [24,30], whereas potential Si–N stretching and bending modes may be overlapped between 950 and 1000 cm^−1^. In addition to these bonding modes, Si–C stretching vibrations are expected between 800–900 cm^−1^ [31,32,33,34,35] to further complicate the deconvolution of the broad band seen in the 800–1100 cm^−1^ range. The vertical lines in Figure 4 illustrate the overlap of the discussed peaks and the qualitative nature of their assignments given the broadness/irregularity of the resulting bands. This kind of data can only be used as supporting evidence for the presence of specific Si–X bond containing species (X = C, N, O), but when supplemented by direct O- and N-content determinations they constitute easily available and relevant information.

A common feature for the powders produced under ammonia is the observation of a relatively broad absorption band centered at 1060–1080 cm^−1^, which is seen to dominate in all these cases. Such a broad band is indicative of absorption environments that have a disruption of long-range order like in amorphous materials/glasses or materials with variations in composition, for instance, those encountered in various tetrahedral groups of the type Si(O_x_,N_y_)_4_ [5,36]. The shoulder at 950 cm^−1^ may be linked to Si–N bending vibrations [37] whereas the band at 470 cm^−1^ is indicative of the Si–O–Si bending mode [38]. As previously discussed, the weak absorption band at 1560 cm^−1^ in the spectra from powders prepared at 1200 °C in ammonia is in the region of the N–H deformation mode in adsorbed NH_3_ as is the medium intensity band peaking at ca. 3370 cm^−1^, that can be attributed to N–H degenerate stretching vibrations. The likelihood that these two bands are due to plausible –NH/NH_2_ terminal groups [39] is rather low given the presence of H_2_O in the gas phase, which would preferentially react with the groups towards Si–OH species.

The FT-IR spectra of the grayish-black colored powders obtained in nitrogen at 1200 °C and 1400 °C show a strongest absorption band peaking at 1080 cm^−1^ assigned to the Si–O–Si stretching mode, a small band at ca. 825 cm^−1^ in the area of the stretching mode of Si–C bonds, and the band at 470 cm^−1^ assigned to the Si–O–Si bending mode. These results suggest that the powders likely contain silica SiO_2_ and some quantities of silicon oxycarbide SiO_x_C_y_ with free carbon, the latter supported by the XRD data and C-content determinations (Table 1 below). Interestingly, the spectra of the powders made at 1600 °C are somewhat different. Besides the wide absorption bands characteristic of the Si–O–Si vibrations, i.e., at 1080 cm^−1^ and 470 cm^−1^, and for the Si–C vibrations at 820 cm^−1^, a distinct new band at ca. 920 cm^−1^ is present that may be assigned to quite strong Si–N modes. The FT-IR results support at most the conclusions derived from the XRD measurements, which confirmed the crystallization of silicon carbide and elemental silicon in both powders. In this regard, crystalline silicon detected by XRD is inactive in infrared spectroscopy. The Si–N bonds supported by FT-IR likely belong to Si(O_x_,N_y_)_4_ structural units in a crystalline silicon oxynitride phase occurring in low enough concentrations below the detection limit of XRD or in the amorphous phase. In this regard, the O- and N-content results show rather low N-contents in those powders (Table 1).

**Figure 5:**
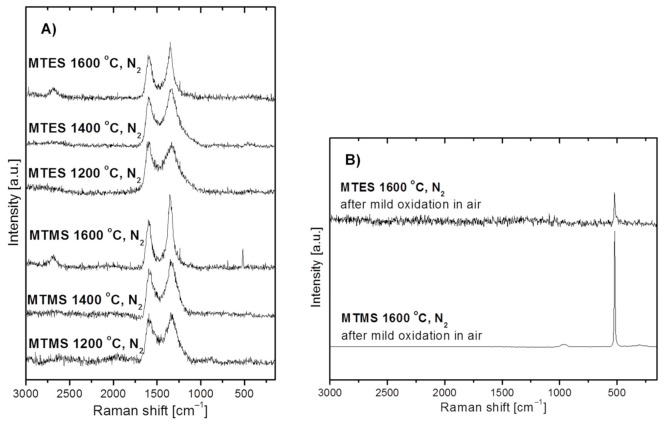
Raman spectra of powders produced by spray pyrolysis from MTMS and MTES in a nitrogen atmosphere. (**A**)—as-produced powders, (**B**)—selected powders after mild oxidation in air.

Figure 5 shows the Raman spectra for the powders produced from MTMS and MTES in nitrogen. Due to free carbon abundance in these powders, all spectra include mainly the characteristic bands related to carbon, whereas the potential bands due to some SiC (expected at ca. 800 and 970 cm^−1^) in the 1600 °C-prepared powders are not discerned at all. Specifically, for all powders the spectra reveal the quite well intensity-balanced bands at 1596 and 1348 cm^−1^ assigned to the so-called G- and D- bands, respectively, which are typical of heat-treated turbostratic carbon materials [40,41,42,43,44,45,46,47,48]. In particular, they are related to the C–C bonding environments typical of, respectively, the sp^2^ and sp^3^ hybridization of carbon atoms or, equivalently, graphite-sheet and diamond-type structure domains. An additional weak and broad band at 2690 cm^−1^, which matches the so-called 2D-band (labelled sometimes as G’), is also observed for both powders produced at 1600 °C. The 2D band is typical for extended graphite sheets with certain thickness, and its presence supports the progressing crystallization of the turbostratic C-domain towards structured graphite, which is consistent with the application of the high temperature of 1600 °C. There is yet another narrow and symmetric band at ca. 520 cm^−1^ for the powder prepared from MTMS at 1600 °C. This peak supports the presence of crystalline silicon (c-Si) since amorphous silicon (a-Si) is characterized by a band shifted to 480 cm^−1^ [49,50]. It is apparent that the applied conditions, mainly the high temperature levels of the order of 1600 °C, favor silicon formation and crystallization. Due to the abundance of carbon in the MTES-derived powder at 1600 °C (see, Table 1 below), no band at 520 cm^−1^ is seen in the original sample being apparently scaled down to the noise level. This is supported by the fact that after the removal of carbon by mild oxidation in air, the weak symmetrical band at 520 cm^−1^ is seen in the respective spectrum, corroborating the XRD-supported presence of a crystalline form of silicon in both products (Figure 5B). A relatively higher intensity band, as expected, is found for the oxidized powder from MTMS. The chemical foundations for elemental silicon formation are described earlier in the section discussing the Raman data. In concluding this section, the Raman spectra for the powders prepared in nitrogen confirm the presence of free carbon as well as some elemental silicon in the 1600 °C-prepared products from both precursor systems. The free carbon components in the composites are characterized by similar proportions of the sp^2^ and sp^3^ C-bonding environments, which is typical for turbostratic carbon. In general, the Raman spectra do not provide any essential information about the silicon-based highly amorphous component(s) of the powders.

Table 1 shows the results of direct O- and N-content as well as free C-content determinations in all powders produced in the study. The highest oxygen levels occur in the powders prepared at the lowest temperature of 1200 °C irrespective of applied gas conditions, whereas the nitrogen content reaches the highest values in the powders made at the highest temperature of 1600 °C. These results suggest that the nitrogen content in the silicon oxynitride-containing powders can be controlled by using appropriate temperature and gas atmosphere conditions. The application of nitrogen gas yields powders containing merely 1–3 wt % of nitrogen, whereas using ammonia results in powders with much higher 14–19 wt % contents of nitrogen. It is instructive to note that these contents are for samples containing carbon and therefore may appear at first glance misleading if the composition of the silicon oxynitride component is referred to. However, under some assumptions the ratio of x for O (at %) and y for N (at %) would provide us with a useful means by which to estimate x and y in SiO_x_N_y_. In this regard, the free C-contents are relatively high for both precursors and, in particular, the contents for the MTES-derived powders are approximately twice as high as those for the MTMS-derived powders. This is consistent with, roughly, this much higher C-content in the initial MTES precursor compared with the MTMS precursor. We also want to point out that the direct O- and N- determinations carried out in a standard way provide with the bulk of the O- and N-contents without specifying surface and/or core-related content contributions that are likely to be very diverse and specific for nanoparticles. In this regard, we have recently been working on a dedicated methodology for the determination in a single run of surface and core-bound oxygen, nitrogen, and hydrogen contents. The data and initial results are soon to be submitted for publication.

Regarding the above, the atomic ratio of O (at %) to N (at %) in the crystalline stoichiometric oxynitride Si_2_ON_2_ is simply 1 to 2 or 0.5. As seen in Table 1, under ammonia and the associated favorable nitridation conditions this ratio is in the range 1.2–2.3 for MTMS and 1.8–2.5 for MTES. The lower the temperature, the higher the ratio, with the latter supporting less efficient nitridation. The best nitridation conditions (with the ratio of O/N equal to 1.2) are found for MTMS processed under ammonia at 1600 °C, whereas at 1200 °C this ratio is approximately twice as high. The numbers for MTMS are generally lower, which is consistent with its better suitability for nitridation compared with MTES. The atomic ratios O/N for powders made in nitrogen are distinctly higher than discussed earlier and confirm a much lower nitridation capability of N_2_ compared with NH_3_. The lowest ratios of 3.7–4.9 are observed here for the 1600 °C-processes. This is yet another manifestation of the efficient carbothermal reduction resulting at this temperature in the formation of the major SiC and elemental Si phases, in addition to some amorphous Si-O_x_N_y_ with the relatively increased oxygen contents.

## 4. Conclusions

The simple and convenient spray (aerosol) pyrolysis synthesis method utilizing two easily available oxygen-bearing organosilicon compounds is shown under selected conditions to yield the highly amorphous silicon oxynitride SiO_x_N_y_ powders in one stage, sometimes in a composite system with free carbon. The latter can be removed by a mild oxidation in air at 700 °C, if necessary, whereas the application of ammonia significantly reduces its quantities. Two chemically different gas atmospheres are applied, namely, a much more reactive ammonia/argon mixture and less reactive nitrogen. The raw products are mostly highly amorphous SiO_x_N_y_ powders (“glasses”) containing some free carbon, with the exception of the process under nitrogen at 1600 °C, where crystalline silicon carbide β-SiC and elemental crystalline silicon (c-Si) are detected in the composite system as well. The products are shown to have diverse chemical compositions dependent on experimental parameters, as supported by direct oxygen and nitrogen content determinations. The highest nitrogen contents in the powders prepared under ammonia are associated with a concomitant decrease in oxygen contents and result from the efficient nitridation of the transient Si–O species towards SiOxN_y_. The use of ammonia is also efficient in the removal of free carbon via the formation of volatile compounds.

The parallelly studied cases of spray pyrolysis of the precursors in nitrogen and in ammonia underline the competing character of the specific reactions there. Thermal decomposition and carbothermal reduction towards SiO_x_C_y_/Si/SiC prevail under nitrogen with a minimal contribution of nitridation. If silicon oxynitride is targeted, the use of a reactive ammonia atmosphere at sufficiently high temperatures promotes the target nitridation reactions towards silicon oxynitride SiO_x_N_y_ and, possibly, silicon nitride Si_3_N_4_ as well.

## Figures and Tables

**Table 1:** The O- and N-contents directly determined in powders produced from MTMS and MTES. The far-right column shows free C-contents determined by mild oxidation in air. Note that O/N ratios are calculated for derived respective atomic percentages.

Precursor	Gas Atmosphere	Temperature [°C]	O Content [wt %]	N Content [wt %]	O/N [at %/at %]	C Free [wt %]
MTMS	NH_3_	1200	39.1	14.7	2.3	<1
		1400	33.6	18.5	1.6	<1
		1600	26.0	19.3	1.2	<1
	N_2_	1200	32.5	1.6	17.8	18.5
		1400	24.4	1.5	14.2	20.5
		1600	16.1	2.9	4.9	21.4
MTES	NH_3_	1200	39.0	13.4	2.5	<1
		1400	35.1	17.1	1.8	<1
		1600	32.9	15.7	1.8	<1
	N_2_	1200	27.0	1.6	14.8	42.8
		1400	27.8	1.5	16.2	40.7
		1600	10.7	2.5	3.7	51.0

## Data Availability

The data presented in this study are available on request from the corresponding author.
